# Spatiotemporal evolution of SARS-CoV-2 in the Bangkok metropolitan region, Thailand, 2020–2022: implications for future outbreak preparedness

**DOI:** 10.1099/mgen.0.001170

**Published:** 2023-12-20

**Authors:** Pakorn Aiewsakun, Bharkbhoom Jamsai, Worakorn Phumiphanjarphak, Waritta Sawaengdee, Prasit Palittapongarnpim, Surakameth Mahasirimongkol

**Affiliations:** ^1^​ Department of Microbiology, Faculty of Science, Mahidol University, Bangkok, 10400, Thailand; ^2^​ Pornchai Matangkasombut Center for Microbial Genomics, Department of Microbiology, Faculty of Science, Mahidol University, Bangkok, 10400, Thailand; ^3^​ Genomics Medicine Center, Medical Life Sciences Institute, Department of Medical Sciences, Ministry of Public Health, Nonthaburi 11000, Thailand

**Keywords:** COVID-19, genomic surveillance, genomic epidemiology, spatiotemporal analysis, Thailand, public health

## Abstract

Thailand experienced five waves of coronavirus disease 2019 (COVID-19) between 2020 and 2022, with the Bangkok Metropolitan Region (BMR) being at the centre of all outbreaks. The molecular evolution of the causative agent of the disease, severe acute respiratory syndrome coronavirus 2 (SARS-CoV-2), has previously been characterized in Thailand, but a detailed spatiotemporal analysis is still lacking. In this study, we comprehensively reviewed the development and timelines of the five COVID-19 outbreaks in Thailand and the public health responses, and also conducted a phylogenetic analysis of 27 913 SARS-CoV-2 genomes from Thailand, together with 7330 global references, to investigate the virus’s spatiotemporal evolution during 2020 and 2022, with a particular focus on the BMR. Limited cross-border transmission was observed during the first four waves in 2020 and 2021, but was common in 2022, aligning well with the timeline of change in the international travel restrictions. Within the country, viruses were mostly restricted to the BMR during the first two waves in 2020, but subsequent waves in 2021 and 2022 saw extensive nationwide transmission of the virus, consistent with the timeline of relaxation of disease control measures employed within the country. Our results also suggest frequent epidemiological connections between Thailand and neighbouring countries during 2020 and 2021 despite relatively stringent international travel controls. The overall sequencing rate of the viruses circulating in the BMR was ~0.525 %, meeting the recommended benchmark, and our analysis supports that this is sufficient for tracking of the trend of the virus burden and genetic diversity. Our findings reveal insights into the local transmission dynamics of SARS-CoV-2 in Thailand, and provide a valuable reference for planning responses to future outbreaks.

## Data Summary

All data analysed in this study are publicly available. Numbers of COVID-19 cases in Thailand by province were obtained from the websites of the Open Government Data of Thailand (https://opendata.data.go.th/dataset/covid-19-daily) and the Department of Disease Control OpenData (https://covid19.ddc.moph.go.th/). Numbers of inhabitants in each province were obtained from the Open Government Data of Thailand (https://opendata.data.go.th/dataset/statbyyear). SARS-CoV-2 genome sequences and their associated metadata were obtained from GISAID (https://gisaid.org/) and Nextstrain (https://nextstrain.org/). R codes used to produce the results in this study are available from https://github.com/PAiewsakun/SARS-CoV-2_in_the_BMR.

**Impact Statement**

This study examines the spatiotemporal evolution of severe acute respiratory syndrome coronavirus 2 (SARS-CoV-2) in Thailand during the five coronavirus disease 2019 (COVID-19) outbreaks between 2020 and 2022, with a specific focus on the Bangkok Metropolitan Region (BMR). The study also reviews the timelines of these outbreaks and the corresponding governmental responses. The overall sequencing rate of the virus within the BMR was estimated at ~0.525 %, meeting the recommended standard for genomic surveillance of SARS-CoV-2 and potential future emerging viruses. Our analysis supports that this sequencing rate is sufficient to track the trend of the virus burden and genetic diversity, and at the same time testifies to Thailand’s capacity to establish an effective genomic sequencing programme for disease surveillance in the future. Our results provide insights into the dynamics of local virus transmission, and the lessons learned from these outbreaks are discussed in the context of enhancing the country’s preparedness for future infectious disease outbreaks.

## Introduction

Thailand was one of the countries that was greatly affected by the coronavirus disease 2019 (COVID-19) pandemic, caused by severe acute respiratory syndrome coronavirus 2 (SARS-CoV-2), an enveloped, positive-sense, single-stranded RNA virus belonging to the genus *Betacoronavirus* within the family *Coronaviridae* and the order Nidovirales [1]. Indeed, Thailand was the first country to report a COVID-19 case outside PR China, reported by the World Health Organization on 13 January 2020 [2]. The outbreaks were most intense in the Bangkok Metropolitan Region (BMR) – an inter-connected and densely populated area of the country, comprising the capital city of Thailand, Bangkok, and five surrounding provinces, Nakhon Pathom, Nonthaburi, Pathum Thani, Samut Prakan and Samut Sakhon. While molecular evolution of SARS-CoV-2 circulating in Thailand in 2020–2022 has already been previously described [3, 4], a comprehensive spatiotemporal analysis of the virus is still lacking.

This study leverages the wealth of whole-genome sequence data of SARS-CoV-2 available on the GISAID and NextStrain databases [5, 6] to investigate spatiotemporal evolution of the virus circulating in the BMR during 2020 and 2022, and to review the development and timelines of the outbreaks and the public health responses. The results provide valuable insights into local transmission dynamics of COVID-19, and are discussed with regard to the preparedness for future disease outbreaks.

## Methods

### Thailand COVID-19 statistics

Numbers of COVID-19 cases in the country by province were obtained from the websites of the Open Government Data of Thailand [7] and the Department of Disease Control OpenData [8]. To estimate province-specific numbers of COVID-19 cases per 100 000, the numbers of inhabitants in each province were obtained from the Open Government Data of Thailand [9]. All data were compiled in October 2022.

### Sequence data collection

We downloaded 13 427 526 SARS-CoV-2 genome sequences from the GISAID database [5] on 10 October 2022, together with their metadata; 12 158 025 were annotated as obtained from original human clinical samples, being more than 27 000 bases long, and had <5 % undefined bases. Of these, 27 913 sequences were annotated as being from Thailand. 13 105 of them were from the BMR; 14 499 were from outside the area, and 309 sequences were of unknown geographical origin.

### Time-calibrated phylogenetic tree reconstruction

To reconstruct the virus phylogeny, a multiple whole-genome sequence alignment of all SARS-CoV-2 samples on the GISAID database was downloaded from the website, and was subsampled to contain only the 27 913 Thai SARS-CoV-2 sequences. By using blastn [10] with an e-value cut off of 1×10^−50^, 4141 non-redundant SARS-CoV-2 sequences originating from outside Thailand on the GISAID database were identified as being highly similar to Thai sequences (top-10 sequences showing blastn best hits), and were readded to the alignment as global reference sequences. The purpose of this global reference collection was to provide context for SARS-CoV-2 circulating in Thailand, particularly in assessing cross-border transmissions. The alignment was further supplemented with 3188 non-redundant pre-aligned sequences sourced from the NextStrain curated global SARS-CoV-2 dataset, focusing on the most recent 6 months of the pandemics up until November 2022, excluding sequences from Thailand. The genome of the Wuhan-Hu-1 sample (GenBank accession number: NC_045512.2), which was one of the earliest samples in the pandemic, was added to the alignment for the purpose of tree rooting. Columns with 90 % gaps or undefined bases in the alignment were removed. The curated alignment contained 35 243 sequences, and 29 711 positions. The accession numbers and metadata for all sequences analysed in this study, including their passage histories, collection dates, PANGO lineage assignments and collection sites, are provided in Table S1, available in the online version of this article. The EPI_SET ID for the GISAID sequences analysed in this study is EPI_SET_23 1130hz (doi: 10.55876/gis8.231130hz).

A maximum-likelihood phylogeny was estimated from the curated alignment using IQ-TREE2 v2.1.3 [11] under the GTR+I+Γ(4) nucleotide substitution model and the fast tree search mode (-fast). The tree was rooted using the Wuhan-Hu-1 sample, and was subsequently time calibrated using lsd2 v2.4.1 [12]. Samples with long terminal branches (*n*=49/35 243=0.139 %, with their root-to-tip distances >0.025 substitutions/site) were removed prior to the time calibration. Short branches were not removed in the analysis, and the variance parameter penalizing long branch lengths was estimated to be 0.000336576 by the program. The temporal constraint that the date of every node must be equal to or smaller than those of its descendants was imposed. The lognormal relaxed clock model (standard deviation=1) was used in the analysis, and the time to most recent common ancestor was constrained to be between 1 December 2019 and 1 January 2020. The reference Wuhan-Hu-1 sample was again used as an outgroup in the analysis and 295 outliers were detected by the program (number of sampling nodes to detect outliers=10 000; Z score threshold to detect outliers=4; estimated median rate=1.19×10^−3^ substitutions/site/year) and were excluded from the analysis (Fig. S1).

### Estimation of the virus effective population size ( Ne ) dynamics

To approximate the Ne dynamic of the viruses in the BMR, we first estimated the overall Ne dynamic of all viruses in the dataset and subtracted that of the viruses collected from outside the BMR, treating the time-calibrated maximum likelihood phylogeny as our best-working hypothesis for the virus evolutionary history. Ne values were estimated in R v4.0.4 using the function mlskygrid in the R package mlesky v0.1.7 [13], with both ‘*res*’ and ‘*tau*’ parameters set to 152, corresponding to the number of epidemiological weeks that our dataset spanned. Parametric bootstrapping (*n*=100) was performed to estimate the uncertainty of the Ne estimates in R v4.0.4 using the function parboot, also from the R package mlesky v0.1.7 [13], with the parameter ‘*dd*’ set to FALSE. Once we obtained the Ne estimates for all viruses in the dataset and for those collected outside the BMR, we then matched them by time, and for each epidemiological week the distribution of the difference between the two Ne estimates was estimated through the Monte Carlo simulation procedure with 100 000 datapoints. The 95 % highest probability density was estimated using the function hdi from the R package HDInterval v0.2.4.

### Estimation of evolutionary rates for variants associated with major outbreaks

Root-to-tip regression analysis was performed to estimate individual rates of evolution of A.6, B.1.36.16, B.1.1.7, AY.30, AY.85, BA.1, BA.2, BA.4 and BA.5, excluding outliers, i.e. samples with atypical branch lengths compared to their collection times (see Methods – Time-calibrated phylogenetic tree reconstruction section – and Fig. S1). For samples with uncertain collection dates, the mid-points of the possible collection date ranges were used in the analysis. The analysis was performed in R v4.0.4 using the function lm.

## Results

### Timeline of the COVID-19 outbreaks in the BMR, 2020–2022

**Fig. 1.**
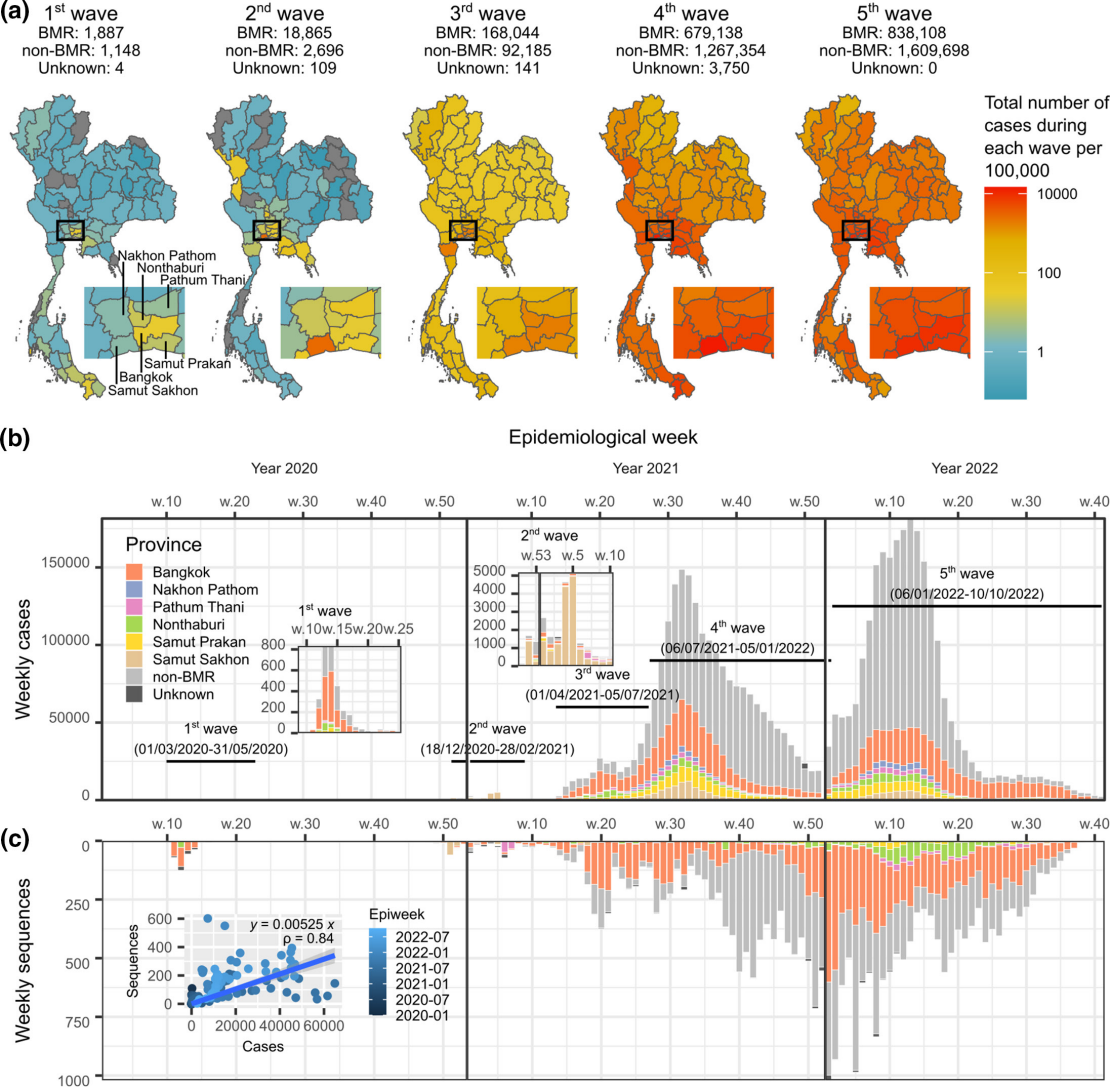
Overview of the five COVID-19 outbreaks in the BMR, 1 January 2020–10 October 2022. (**a**) Maps of cumulative COVID-19 cases per 100 000 inhabitants during each COVID-19 wave in Thailand. Numbers of the reported COVID-19 cases in each wave from the BMR, outside the BMR, and those from unknown regions are shown on top of the maps. Boxes indicate the BMR. Inset: maps of the expanded view of the BMR. (**b**) Weekly COVID-19 cases in Thailand 1 January 2020–10 October 2022. The chart is coloured according to province, with the six provinces in the BMR showing distinctive colours (see key). Time frames of the five waves of COVID-19 in the country are indicated. (**c**) Weekly SARS-CoV-2 sequences from Thailand made publicly available on the GISAID database [5] collected 1 January 2020–10 October 2022. Inset: numbers of weekly COVID-19 cases in the BMR and respective SARS-CoV-2 sequences show a positive linear correlation (blue line).

Between 2020 and 2022, the BMR saw a total of five waves of COVID-19; each was larger than the previous one (Fig. 1a, b). Over the course of the five waves, the country saw more than 4.68 million cases of COVID-19, and more than 32 000 deaths resulted from the infection [14]. The BMR alone saw 1 706 042 cases of COVID-19, accounting for 36.46 % of the total confirmed cases in the country (the rest of the country: 2 973 081 cases, 63.54 %, Fig. 1a). This indicates that the BMR was indeed the epicentre of all five COVID-19 waves. Government reports and various news sources had shed light on the unique characteristics of each wave. Brief reviews of the outbreak timelines and government responses during the five waves are provided below.

### First wave

After the first reported case in January 2020 [2], Thailand saw a total of 42 COVID-19 cases by the end of February 2020 [15], most of which were imported cases and did not cause widespread outbreaks. However, during mid-March and April 2020, Thailand saw a rapid surge in the number of COVID-19 cases throughout the country, making it one of the first countries to experience a large COVID-19 outbreak outside PR China, highlighting it as a hotspot for emerging infectious diseases. Contact tracing suggested that the outbreak started with two super-spreading events tracing back to a Thai boxing stadium and nightlife entertainment venues in Bangkok, and the virus was determined to be of the S-clade variant (PANGO lineage: A.6) [16, 17].

In response to the emerging outbreak, the Thai Government declared a state of emergency, and implemented multiple highly stringent disease control measures nationwide. This included a full-scale national lockdown, bans of public gatherings, 10 pm-to-4 am curfews, cross-provincial travel bans, mandated temporary closure of various major public venues and business establishments within the BMR, as well as 14 day mandatory quarantine for international travellers [18]. These highly restrictive control measures were effective, but also brought about serious negative social and economic impacts to the country [18]. The outbreak eventually subsided in May 2020 [16, 17], concluding the first wave of COVID-19 in Thailand (between 1 March 2020–31 May 2020, Fig. 1a). Easing of restrictions was gradually implemented afterward [19], and the curfew was lifted in mid-June [20].

### Second wave

After almost half a year of no local COVID-19 transmissions, Thailand saw an resurgence of COVID-19 cases once again in December 2020, marking the beginning of the second wave of COVID-19 within the country [21]. The second wave started from 18 December 2020, with its epicentre identified in Samut Sakhon, a province within the BMR, and home of a large number of Myanmarese migrant workers [22]. Most of the early reported cases during the second wave were Myanmarese migrants who lived in Samut Sakhon and Pathum Thani, another province in the BMR [22]. The dominant variant was determined to be the GH-clade variant (PANGO lineage: B.1.36.16). This variant was highly prevalent in Myanmar at the time, and it was suggested that the virus might have come from Myanmar [21].

Several disease control measures were implemented during this wave, but not as stringent as in the first one to avoid adverse socioeconomic disruptions. During the second wave, the government mandated the temporary closure of schools for the entire month of January, and recommended suspension of businesses and crowded activities in some specific areas that experienced high incidences of COVID-19 [23]. The government also employed an active-case finding strategy, and strategically mobilized essential medical resources, including case-finding teams and clinicians, to the areas in need [21]. With all of these concerted efforts, the country was able to contain the outbreak by February 2021 [21]. Most of the cases during this wave were young healthy working-group adults, and while the total COVID-19 cases in the second wave (21 670 cases, 18 December 2020–28 February 2021, Fig. 1a) far exceeded those of the first one (3 036 cases), most of the infections were found to be asymptomatic, and the case fatality rate was much lower in comparison (0.11 vs 1.46 %) [21]. A COVID-19 vaccination programme (the inactivated vaccine CoronaVac, Sinovac Biotech, and the viral vector vaccine ChAdOx1-nCoV, AstraZeneca) was initiated towards the end of the second wave in late February 2021, with the primary targets being healthcare workers, elderly people and people with congenital diseases [24].

### Third wave

A few months after the second wave, Thailand encountered yet another wave of COVID-19 in April 2021. The third wave was determined to involve a more contagious variant of SARS-CoV-2 compared to the first two, namely the alpha variant (PANGO lineage: B.1.1.7). The early upsurge of the virus was linked to nightlife entertainment venues in Bangkok [25], before it was found to subsequently spread to other local communities, and eventually all over the country. The country saw four-figure new daily cases for the first time during this wave, which peaked on 17 May 2021 at 9635 new daily cases per day [26].

In response to the escalating situation, the government employed multiple disease control measures, including temporary closure of entertainment venues and schools in Bangkok in April [27], and cancellation of the traditional Thai New Year (Songkran) festival, which was also to occur in April [28] (although without a city lockdown and cross-provincial travel bans [29]). Field hospitals were set up to facilitate patient isolation at the time [27]. The mass national vaccination programme (CoronaVac, Sinovac Biotech, and ChAdOx1-nCoV, AstraZeneca) was rolled out in June 2021 to de-escalate the situation, but again with a limited supply [30].

Amidst the third wave, the delta variant (PANGO lineages: B.1.167.2 and AY lineages) made inroads. The virus was more transmissible than the alpha variant [31–33], and rapidly established itself as the dominant variant among new COVID-19 cases. This eventually led to the fourth wave of the COVID-19 epidemic in Thailand, starting from 6 July 2021. The third wave concluded with 260 370 cases of COVID-19 patients nationwide (1 April 2021–5 July 2021, Fig. 1a).

### Fourth wave

Local transmission of the delta variant was detected for the first time in the country in late May 2021 among construction workers in Bangkok [34], approximately 1 month before formal recognition of the fourth wave. The number of delta cases increased very rapidly, and by mid-July 2021 63 % of newly infected cases in the country were found to be of the delta variant, while the alpha variant accounted for only 34 % of new cases. It was also during this period that the country saw the number of new daily cases surpassing five figures for the first time [35].

In response to the rapidly escalating situation, the government imposed multiple disease control measures on the entire BMR and several other provinces that showed rising trends in COVID-19 cases starting from 19 July 2021, including city lockdown [36]. Concurrently, the mass national vaccination programme with CoronaVac and ChAdOx1-nCoV vaccines continued. In late July to early August 2021, an mRNA COVID-19 vaccine (BNT162b2, Pfizer–BioNTech) became available in the country, but with limited supply, and was mostly available to healthcare workers [37]. The number of daily new cases and hospitalizations continued to rise rapidly, and reached a peak in mid-August 2021, with the maximum daily cases of 22 782 reported on 12 August 2021 [38]. During this intense period, more than 200 000 COVID-19 patients were hospitalized in the public healthcare system at the same time [38, 39], placing an immense strain on healthcare facilities nationwide. The outbreak, however, eventually began to decline starting from late August 2021 onwards [40]. In October and November 2021, two mRNA COVID-19 vaccines, BNT162b2 (Pfizer–BioNTech) [41] and mRNA-1273 (Moderna) [42], became available to the wider Thai population through the national vaccination programme and private hospitals. The number of daily cases dropped below 9000 for the first time since early June in late October 2021 [43], and in November 2021 the government decided to reopen the country to foreigners from 63 countries without mandating quarantine [44]. By that time, 42 million people in the country had received at least one dose of COVID-19 vaccination, covering 60 % of the Thai population and 85 % of the government target [45]. The fourth wave continued to show a steady downward trend both in terms of numbers of new daily cases and hospitalized cases until December 2021 [46, 47], when the omicron variant made an appearance in the country.

### Fifth wave

As the fourth wave wound down, the omicron variant (PANGO lineages: B.1.1.529 and BA lineages) was detected for the first time in Thailand among cross-country travellers in mid-December 2021 [46], and local omicron community transmissions were reported from many regions of the country by the end of the year [47]. By 6 January 2022, Thailand saw omicron infections in the majority of the provinces [48], marking the beginning of the fifth wave [49]. From January 2022 onwards, new daily cases continued to rise once again, and reached a peak during the first week of April 2022, with a maximum daily case count of 25 863, averaged between 31 March and 6 April 2022 [50]. After the first week of April 2022, the epidemic started to show a steady downward trend. Although the fifth wave lasted for longer, and the overall case count was much greater (2 447 806 cases, 6 January 2022–10 October 2022, Fig. 1a) compared to the fourth one (1 950 242 cases, 6 July 2021–5 January 2022, Fig. 1a), the total number of deaths during the fifth wave was markedly lower [total COVID-19-related reported deaths: fifth wave, 11 069 (0.45 % case fatality rate); fourth wave, 39 147 (2.01 % case fatality rate)]. This finding aligns well with the established fact that omicron variants generally induce milder symptoms compared to the delta variants [51].

Throughout these five outbreaks, extensive nationwide efforts were undertaken to sequence SARS-CoV-2 samples in Thailand to aid transmission investigations, contact tracing and monitoring of new virus variants. These concerted efforts resulted in a large collection of whole-genome sequences of SARS-CoV-2 from the country, made publicly available through the GISAID database [5].

### Genome sequence lineage diversity

As of 10 October 2022, the GISAID database had 30 566 SARS-CoV-2 genome sequences annotated as originating from Thailand. Among these, 27 913 were annotated as derived from original human clinical samples, containing more than 27 000 bases, and having <5 % undefined bases (Fig. 1c). In line with the fact that the BMR was the epicentre of all of the five COVID-19 waves, almost half of the sequence collection was reported as being from the BMR (13 105/27 913=46.95 % of the Thai sequence collection). The majority of the BMR sequences were from Bangkok (10 729/13 105=81.87 % of the BMR sequence collection), followed by Nonthaburi (1373, 10.48 %), Pathum Thani (439, 3.35 %), Samut Prakan (326, 2.49 %), Samut Sakhon (206, 1.57 %) and Nakhon Pathom (32, 0.24 %). The rest were either from other provinces (14 499/27 913=51.94 %) or had an unknown geographical origin at the provincial level (309/27 913=1.11 %). The numbers of weekly reported COVID-19 cases in the BMR and the SARS-CoV-2 genome sequences originating from the region showed a strong positive correlation (Spearman correlation=0.84; Fig. 1c, inset graph), indicating that the data were a good temporal representation of the virus outbreaks. The sequencing rate in the BMR was estimated to be ~5.25×10^−3^=0.525 % based on a linear regression analysis (Fig. 1c, inset graph), comparable to the previous nationwide estimate of 0.55 % [52].

**Fig. 2.**
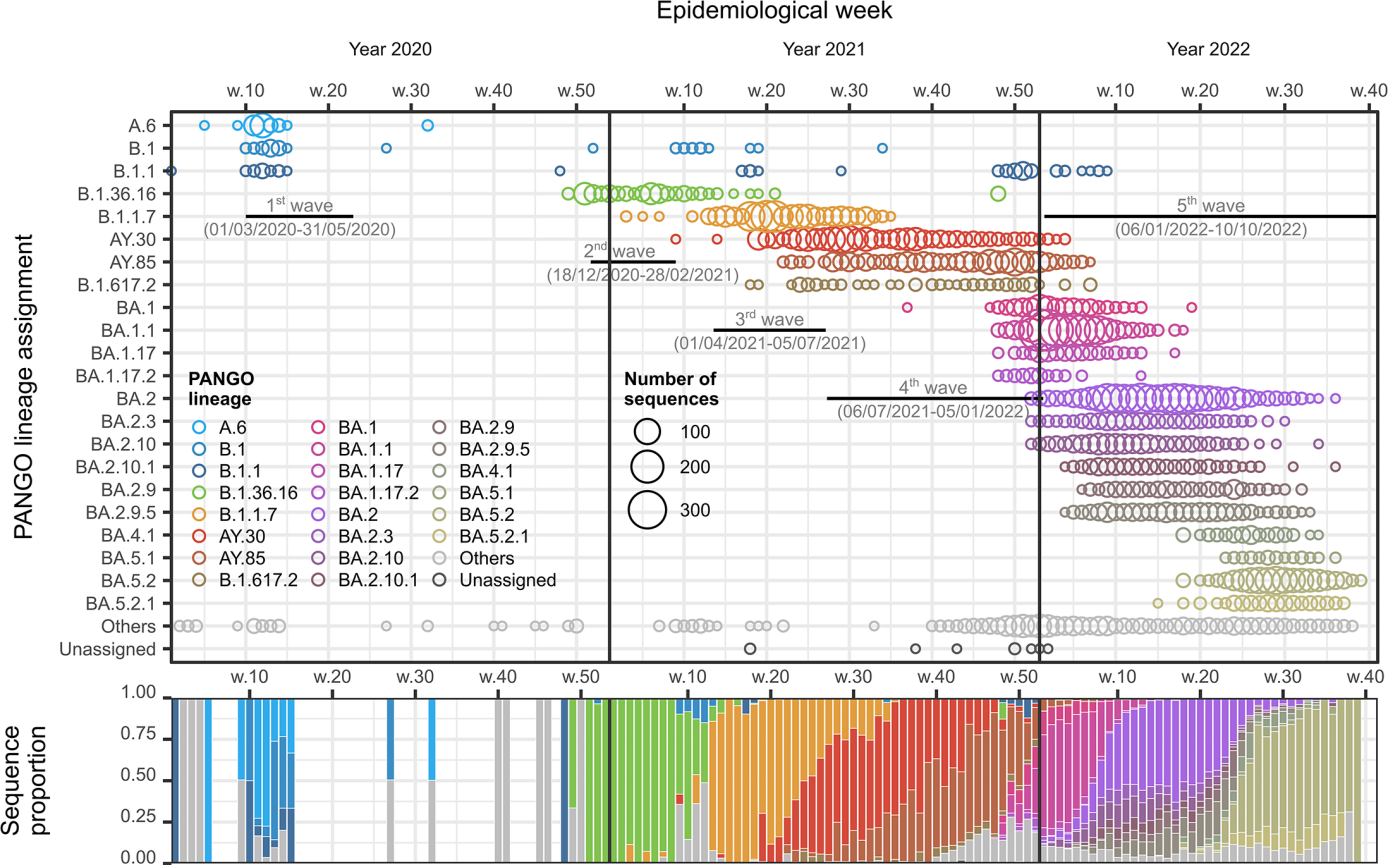
SARS-CoV-2 sequences by variant, BMR, Thailand, 1 January 2020–10 October 2022. Time frames of the five waves of COVID-19 in the country are indicated. The vertical orders of PANGO lineages in both the bubble chart (top) and the stacked bar chart (bottom) are the same.

Sequences originating from the BMR (*n*=13 105) were assigned to 234 different PANGO variants. Among these, we identified 22 variants as being ‘predominant’ variants, defined in this study as having more than 30 sequences and constituting more than 5 % of the sequence data in at least one epidemiological week during the epidemics. These variants included A.6, B.1., B.1.1, B.1.36.16, B.1.1.7, B.1.617.2, AY.30, AY.85, BA.1, BA.1.1, BA.1.17, BA.1.17.2, BA.2, BA.2.3, BA.2.9, BA.2.9.5, BA.2.10, BA.2.10.1, BA.4.1, BA.5.1, BA.5.2 and BA.5.2.1 (Fig. 2, top). The variant B.1.617.2 corresponds to the classic delta variant, and the variants AY.30 (also known as B.1.617.2.30) and AY.85 (B.1.617.2.85) are two distinct variants evolving from the classic delta. All BA variants are subvariants of the B.1.1.529 (omicron) variant. The dominant sequence variants during each wave are largely consistent with the reports from the government and various new sources outlined above. The classic lineage turnover cycles could be clearly observed (Fig. 2, bottom).

### Phylogenetic analysis

**Fig. 3.**
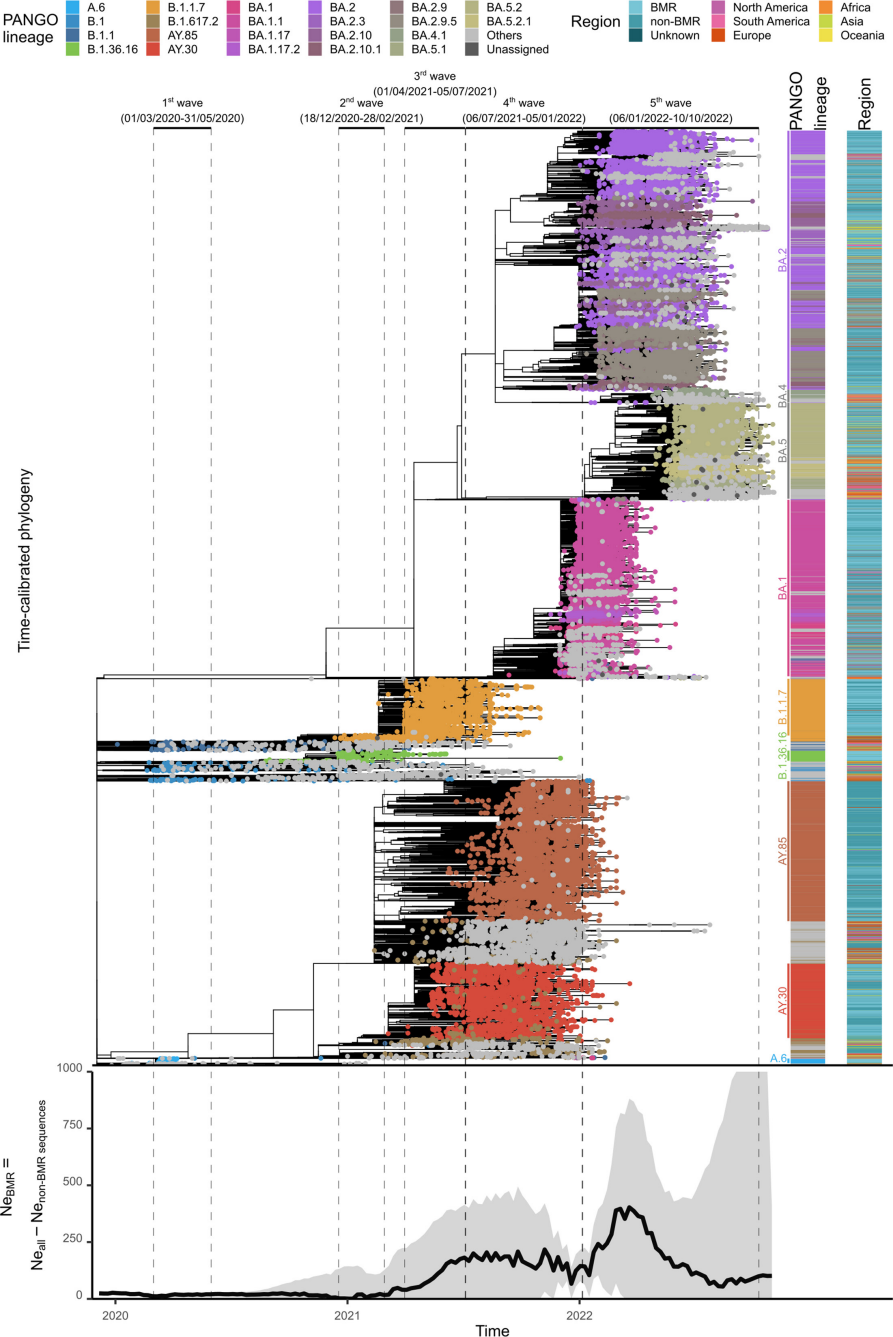
Top: time-calibrated phylogeny of SARS-CoV-2 in the BMR (*n*=13 005) against a backdrop of non-BMR sequences from Thailand (*n*=14 720) and global reference sequences (*n*=7173), and (bottom) their effective population size ( Ne ) dynamic. Time frames of the five waves of COVID-19 in the country are indicated. The Ne dynamics of the viruses in the BMR was estimated by estimating the Ne dynamic of all viruses in the dataset and subtracting that of the viruses from outside the BMR. Solid black line indicates the median estimate and the grey shading area indicates the 95 % highest probability density. The graph depicts Ne values between 0 and 1000 only.

Tip-dating phylogenetic analysis was performed to examine the phylogenetic structure of SARS-CoV-2 within the BMR (*n*=13,105) against a backdrop of 14 808 non-BMR sequences from Thailand and 7329 global reference sequences that showed high sequence similarity to Thai sequences. The Wuhan-Hu-1 sequence, one of the earliest sequences reported in the pandemic, was used to as an outgroup. One hundred BMR sequences, 88 non-BMR sequences and 156 non-Thai sequences were detected as having atypical branch lengths compared to their collection times, and were thus excluded from the analysis (Fig. S1). Our analysis yielded a median evolutionary rate of 1.19×10^−3^ substitutions/site/year (s/n/y), comparable to previous estimates of 1.24–2.60×10^−3^ s/n/y [3, 4], and viruses of the same major variants formed their own distinct clusters (Fig. 3, top). Detailed descriptions of the phylogenetic structures of individual variants dominating each wave (first: A.6, B.1 and B.1.1; second: B.1.36.16; third: B.1.1.7; fourth: B.1.617.2, AY.30 and AY.85; and fifth: various BA.1, BA.2, BA.4 and BA.5 subvariants) are provided in ์Notes S1.

In summary, we found that Thai A.6 and B.1.36.16 sequences formed their own distinct clusters, most of which were sequences from the BMR (Figs 2 and 3 and Notes S1). Thai B.1 and B.1.1 sequences collected during the first wave were found to be clustering with those reported from other countries but without forming large clusters (Fig. S2). These likely represented sporadic imported cases. In contrast, there were a large number of Thai B.1.1.7 sequences that were reported from outside the BMR, and these formed a mixed phylogenetic cluster with the BMR sequences (Fig. S4). A similar pattern was observed for AY.30 and AY.85 (Fig. S5). These suggested that cross-provincial transmissions were common and extensive in Thailand during the third and fourth waves. Notably, AY.30 and AY.85 formed their own distinct clusters, suggesting that they were introduced to the country independently. Interestingly, we also found that the Thai BMR sequence collection has a higher proportion of AY.30 sequences (56.4 %, 95 % confidence interval=54.3–58.4 %) compared to the Thai non-BMR sequence collection (24.3%, 95 % confidence interval=23.1–25.4 %), and the difference was significant [type III analysis of variance (ANOVA) test: df=1, χ^2^=725.47, *P*<2.2×10^−16^]. This finding indicates differential spatial distributions of the two viruses, and their potentially different transmission routes.

We found that clusters of the Thai A.6, B.1.36.16, B.1.1.7, AY.30 and AY.85 sequences contained relatively low numbers of sequences from other countries (Figs S2–S5), suggesting limited cross-border virus transmission during the first four COVID-19 waves in Thailand. However, our analysis still detected sequences from a few countries exhibiting high similarity and clustering with Thai sequences towards the base of the clusters, including sequences from Myanmar, Hong Kong, Singapore, The Republic of Korea, United Arab Emirates and a few European countries, but most commonly Cambodia (in all four waves). This suggests that there were epidemiological connections between Thailand and these countries during the early stages of the first four waves, but most strongly with Cambodia, despite strong international travel controls being imposed at the time. Furthermore, our analysis revealed that the times to most recent common ancestors of these viruses, which were mainly restricted to Thailand, often proceeded their earliest reported sequences by several months (Notes S1). This suggests a possibility that the viruses could have emerged, and potentially circulated within the country, for several months before being observed/causing large outbreaks.

In contrast, sequence clusters of the BA.1, BA.2, BA.4 and BA.5 omicron variants, which drove the fifth wave of COVID-19 in Thailand in 2022, contain large numbers of both Thai and non-Thai sequences, as well as sequences from the BMR and other regions of Thailand, intermixing together phylogenetically (Fig. S6 and Notes S1). This pattern supports the view that the fifth wave was a result of multiple independent virus introductions into the country and extensive local transmissions.

**Fig. 4.**
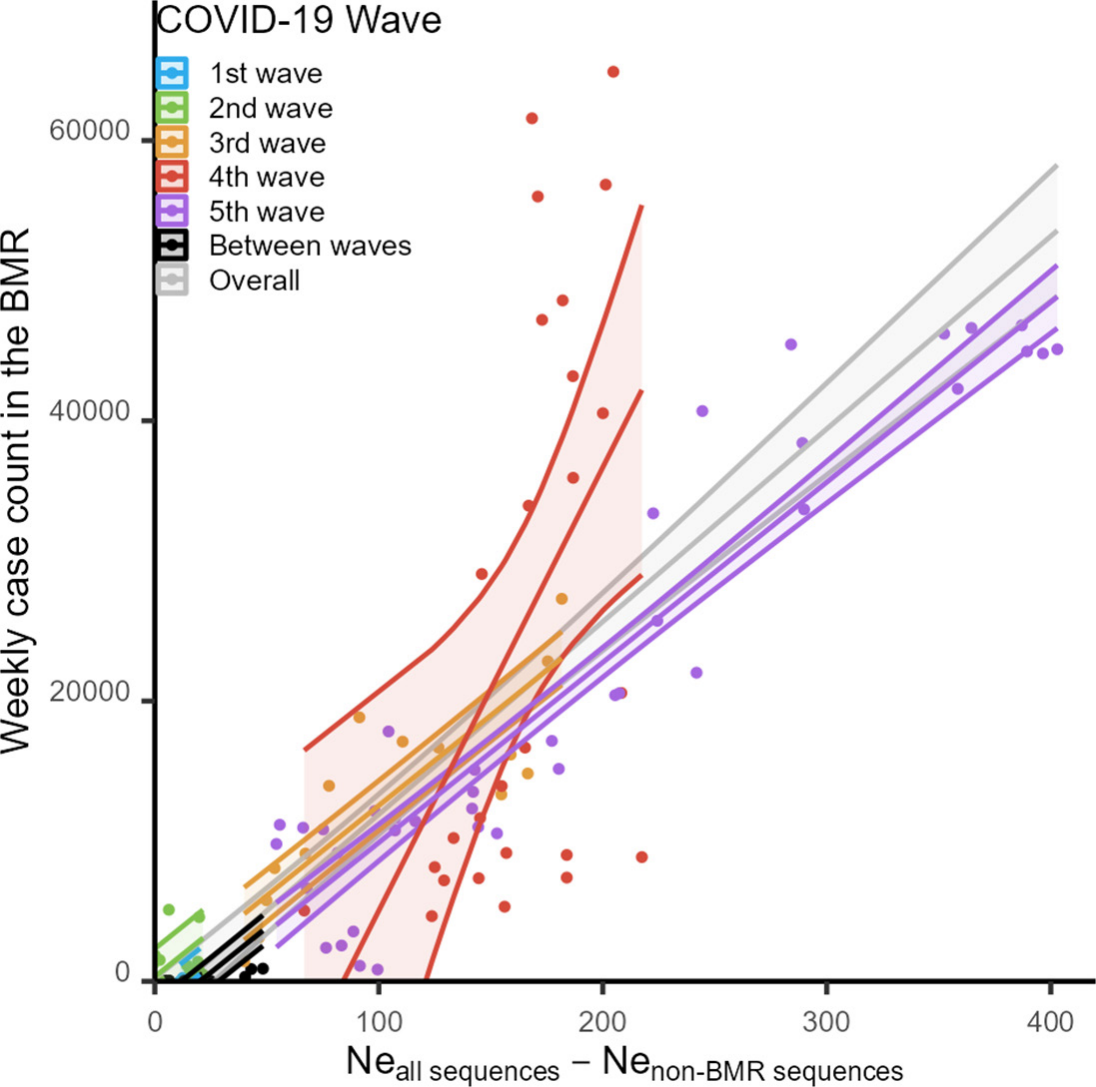
Linear correlation between the median Ne estimates of the viruses in the BMR and the reported weekly COVID-19 case counts.

The dynamic of the effective population size ( Ne ) of the viruses in the BMR was also estimated from the tree obtained (Fig. 3, bottom), showing an overall strong and robust linear relationship with the numbers of reported weekly COVID-19 cases (Fig. 4; type III ANOVA test: slope=137.532, df=1, F=335.136, *P*<2×10^−16^, R^2^=0.702). The slopes of the regression lines across all waves, except for the fourth one, were not significantly different form one another (type III ANOVA test: df=4, F=1.109, *P*=0.356), and the model showed a good fit to the data (R^2^=0.941, type III ANOVA test: slope=128.597, df=1, F=760.1878, *P*<2×10^−16^). The slope of the fourth wave was notably greater than those of other waves (type III ANOVA test: slope=316.400, df=1, F=8.485, *P*=0.0076, R^2^=0.261).

### Evolutionary rates of SARS-CoV-2 variants causing major outbreaks in the BMR and Thailand

**Fig. 5.**
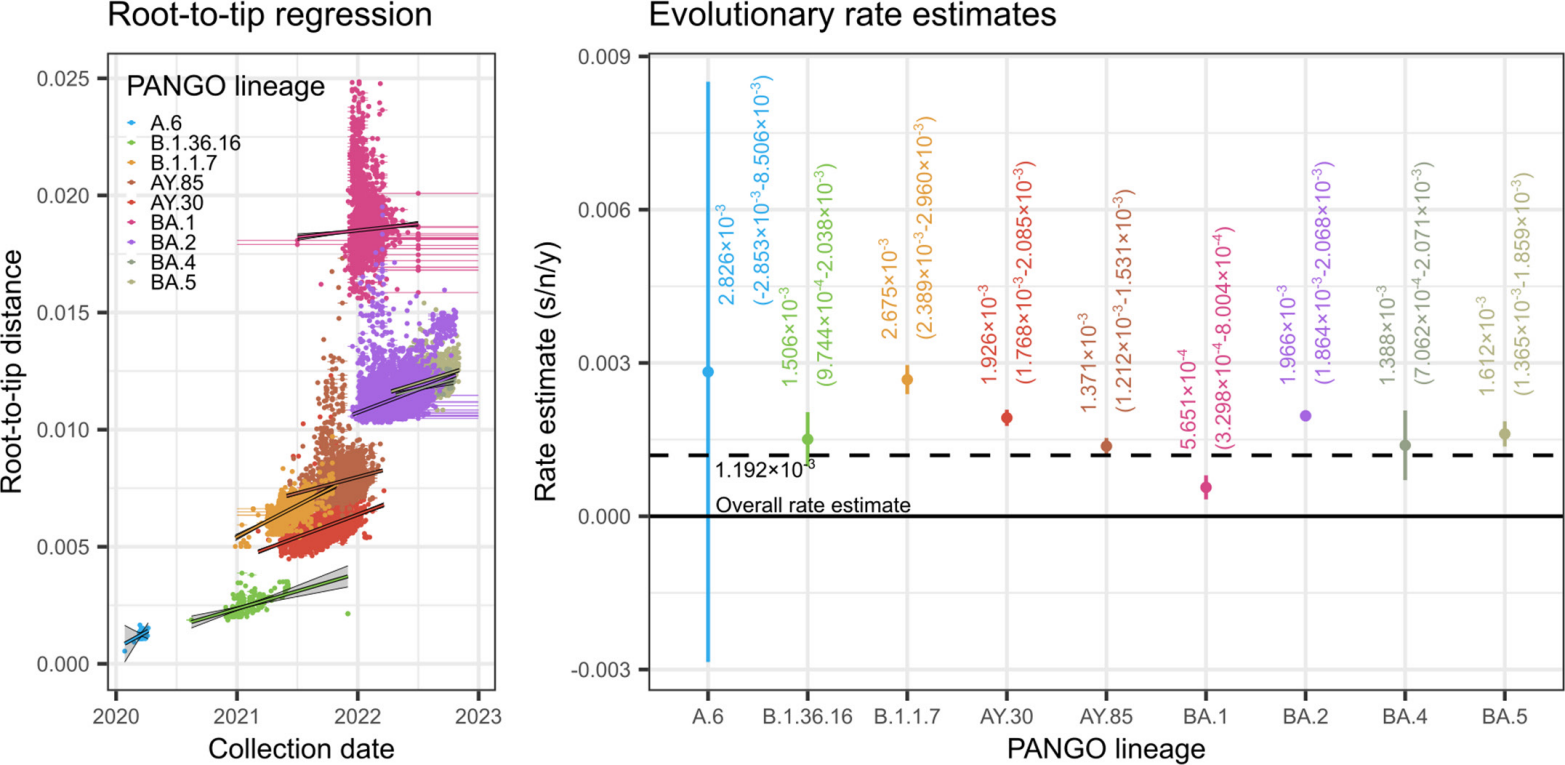
Root-to-tip regression analysis of various SARS-CoV-2 variants causing major outbreaks in the BMR and Thailand. Left: separate slopes and intercepts were estimated for each individual variant, including A.6, B.1.36.16, B.1.1.7, AY.30, AY.85, BA.1, BA.2, BA.4 and BA.5, excluding outliers, i.e. samples with atypical branch lengths compared to their collection times (see Methods and Fig. S1). Horizontal lines indicate collection date uncertainties. For samples with uncertain collection dates, the mid-points of the date range were used in the analysis. Best-fit linear models (solid lines) and their regression confidence intervals (grey shading areas) are shown. Right: evolutionary rate estimates for the nine virus variants (i.e. the estimated slopes of their root-to-tip regression models). Vertical lines indicate 95 % confidence intervals. Horizontal dotted line indicates the overall rate estimate.

The mean rates of evolution of individual variants associated with major outbreaks in the BMR and Thailand, including A.6, B.1.36.16, B.1.1.7, AY.30, AY.85, BA.1, BA.2, BA.4 and BA.5, were estimated using root-to-tip regression (Fig. 5). Temporal signals in the sequence data of all individual variants were sufficient for robust rate estimations, with the exception of A.6 (*n*=170), which were collected over a short time frame (27 January 2020–7 April 2020, 72 days), and thus may lack sufficient evolutionary changes to produce a meaningful and robust rate estimate. For variants with meaningful rate estimates, they all have generally comparable rates of evolution, varying by no more than 2.5 times around the overall rate, ranging from 5.651×10^−4^–2.675×10^−3^ s/n/y (Fig. 5, right). This aligns with a previous finding that local rates of evolution for variants of concern do not differ significantly from the overall background rate [53].

Another notable trend in the results is the upward shift on the *y*-axis of the root-to-tip regression models for variants emerging later in time compared to earlier variants (Fig. 5, left). In particular, the regression lines of B.1.1.7, AY.30 and AY.85 are notably higher than those of A.6 and B.1.36.16, and those of the various BA sub-omicron variants are the highest of all. This pattern indicates accelerated evolutionary rates on the stem branches connecting these variants to the background, again in line with a previous finding [53].

## Discussion

The global surveillance and analysis of SARS-CoV-2 genome sequences have proved to be instrumental in tracking the spread of the virus, understanding its evolutionary path, identifying newly emerging variants and guiding appropriate public health responses. This study analysed publicly available SARS-CoV-2 genome sequences to investigate the spatiotemporal evolution of the virus in Thailand from 2020 to 2022, with a specific focus on the BMR, and reviewed the situations at the time. The analysis identifies dominant virus variants that were circulating in the country during each COVID-19 wave, and provides valuable insights into the dynamics of the virus local transmission. Our findings also provide valuable reference for the formulation of effective responses to future outbreaks.

Our study confirms the BMR as the epicentre of COVID-19 in Thailand across all five waves of the disease (Fig. 1). This is perhaps unsurprising given that the BMR is an international hub and the centre of socioeconomic activity in the country, and underscoring it as a region that should be monitored closely during future disease outbreaks. The dominant variants responsible for major outbreaks during the first to fifth waves were A.6, B.1.36.16, B.1.1.7, two-sub-delta variants (namely AY.30 and AY.85), and several sub-omicron variants (BA.1, BA.2, BA.4 and BA.5), respectively (Fig. 2, top). Their individual rates of evolution are comparable, ranging between 5.651×10^−4^ and 2.675×10^−3^ s/n/y (Fig. 5, right). Nevertheless, shifts in dominant variants could still be clearly observed (Fig. 2, bottom), indicating strong local competition, and the successive gain of competitive advantage and improved transmission capacity of the virus over time. In this regard, it has been suggested that the emergence of new and successively more competitive SARS-CoV-2 variants is driven by accelerated accumulations of evolutionary changes on their stem branches, showing rates around four times faster than the background one [53]. The signature of this phenomenon (i.e. the upward shift on the *y*-axis of the root-to-tip regression models for later variants compared to earlier ones) can also be seen clearly in this study (Fig. 5, left), aligning well with a previous report [53].

It is also worth noting that, just before the onset of the fifth wave in November 2021, 42 million people in the country were reported to have received at least 1 dose of COVID-19 vaccination already, covering 60 % of the Thai population and 85 % of the government target [45]. Despite this substantial vaccine coverage, the fifth wave still occurred in 2022 with the highest new daily case record, driven by various sub-omicron variants. This strongly suggests that the viruses were capable of escaping immunity induced by the COVID-19 vaccines available at the time, as previously reported [54–56]. Together, these observations highlight the rapid evolutionary dynamics of the virus, and underscore the importance of timely genomic surveillance to detect genetic changes that might affect the transmissibility and immune evasion of the virus, as well as its virulence and pathogenesis.

Overall, based on the sequences made publicly available on the GISAID database, we estimated the sequencing rate of the viruses circulating in the BMR to be ~0.525 % (Fig. 1). This estimate is comparable to the nationwide estimate of 0.55 % previously reported by Brito *et al*. [52], and met their recommended benchmark of 0.5 % for genomic surveillance of SARS-CoV-2 and potential future emerging viruses. Our analysis revealed that the Ne dynamics of the viruses estimated from this sequence collection showed a close correspondence with the recorded weekly case counts (Fig. 4), supporting the view that a 0.5 % sequencing rate could indeed be sufficient for monitoring of the trend of viral transmission and genetic diversity. This also testifies to Thailand’s capacity to establish an effective genomic sequencing programme for surveillance of infectious diseases in the future.

Our analysis suggests that the observed COVID-19 outbreaks during the first four waves in Thailand, 2020–2021, were a result of a limited number of virus introductions to the country, and the spread of the virus were mainly driven by local transmissions (Figs S2–S5 and Notes S1). This period aligns well with the time when many countries imposed stringent restrictions on international travel to mitigate cross-border COVID-19 transmission, including Thailand (see Results – Overview of the COVID-19 outbreaks in the BMR between 2020 and 2022). Indeed, it was reported that global international travel decreased by 72 % in 2020, and 71 % in 2021 compared to the pre-pandemic levels in 2019 [57], explaining the situations. In addition, we found that, as of the time of this analysis, A.6, B.1.36.16, AY.30 and AY.85 sequences from Thailand accounted for a large portion of the sequences on the GISAID database [94.74 % (216/288), 49.82 % (414/831), 72.51 % (2777/3830) and 87.47 % (5383/6154), respectively]. This indicates that these variants were indeed primarily restricted to Thailand, in line with the findings discussed above. On the other hand, there were 1 176 623 B.1.1.7 sequences on the GISAID database, and only 2201 of them were reported from Thailand (0.187 %). However, it is clear from our analysis that there are low numbers of B.1.1.7 sequences from other countries that exhibited high similarity to Thai sequences, and Thai B.1.1.7 sequences formed a distinct phylogenetic cluster separate from those reported from other countries (Fig. S4). This suggests the existence of a distinct B.1.1.7 sub-lineage that was relatively unique to Thailand, but the classification resolution of the variant was simply low, so that geographical restriction of the virus could not be observed at the PANGO lineage level.

Despite the limited cross-border virus transmission observed during 2020 and 2021, our analysis detected sequences from a few neighbouring countries that were often found to cluster with Thai sequences, in particular Cambodia. This implies a strong epidemiological link between the two countries during the first four waves despite strong international travel controls in place. In addition, our phylogenetic analysis also suggested that the second wave of COVID-19 in Thailand likely linked with the outbreak in Myanmar during that time (Fig. S3, and Notes S1), consistent with a previous suggestion made based on epidemiological profiles of the virus and the travel histories of the patients [21]. Immigrant people from these two countries indeed contribute significantly to the workforce of Thailand. In 2019, Plan International Thailand reported that Thailand was the most popular overseas destination for both Myanmarese and Cambodian workers, with ~3.09 million Myanmarese individuals and 1.70 million Cambodians estimated to be working in Thailand [58]. This finding emphasizes the need for improved health screening measures for immigrant workers in future outbreaks.

Moreover, our analysis of sequences collected during the first four waves revealed that new virus variants could potentially emerge and circulate for several months before being observed/causing large outbreaks (Notes S1). Rapid detection of new variants is essential for timely adaptation of public health responses and disease control measures. In terms of preparedness for future outbreaks, this highlights the necessity for Thailand to invest in advanced genomic surveillance infrastructure and foster collaborations with other countries, especially neighbouring ones, to detect newly emerging pathogens more rapidly. This also underscores the need for rapid communication channels to facilitate more timely dissemination of critical genomic data. This proactive approach is essential when dealing with rapidly evolving pathogens such as viruses, and by doing so, Thailand can respond more effectively to future disease outbreaks.

In contrast to the dynamics observed during the first four COVID-19 waves, our analysis detected a large set of diverse sequences from other countries showing high similarity to Thai omicron sequences collected during the fifth wave. These sequences formed mixed phylogenetic clusters together (Fig. S6), suggesting that the fifth wave, which began in early 2022, was a result of multiple independent introductions of the virus into the country, and cross-border transmissions were common. This time period coincided with when Thailand reopened the country to foreign travellers without mandatory quarantine in late 2021, and it was reported that international tourism in 2022 doubled that of 2021 [57], providing a plausible explanation for the observed shift in the epidemiological dynamic.

Focusing on local virus transmissions, our results show that the outbreaks during the first two waves were mainly localized within the BMR (Figs 1, S2 and S3). Consistent with this, both the total reported cases (Fig. 1) and the estimated Ne values (Fig. 3) were also relatively low during these periods. This suggests that the disease control measures and responses implemented at the time were highly effective. However, the measures used were stringent, particularly during the first wave, including full-scale lockdowns, bans on public gatherings, curfews and mandated temporary closure of major public and business venues. Given that the BMR is the socioeconomic centre of Thailand, these also resulted in significant economic disruptions for the entire country.

Learning from the first year of the pandemic, the travel restrictions and disease control measures employed by the government to mitigate virus transmission in the second and third year of the pandemics were not as stringent to avoid severe economic disruptions. The general Thai population also learned to became more accustomed to the disease. Perhaps as a consequence of this, we found that virus transmissions between the BMR and the rest of the country became very common during the third, fourth and fifth waves (Figs S4–S6 and Notes S1). This is also reflected in both the total reported cases (Fig. 1) and the estimated Ne values (Fig. 3), which were substantially higher in comparison to the first two waves. For future outbreaks, it is thus very important to consider a balanced approach when planning disease control measures and responses, particularly in regions like the BMR, where the delicate balance between curbing disease transmission and minimizing adverse socioeconomic impacts must be carefully managed.

Interestingly, our analysis revealed that there were at least two large separate outbreaks that were cooccurring during the fourth wave of COVID-19. These outbreaks involved two distinct sub-delta variants, AY.30 and AY.85, which were introduced into the country independently. The BMR sequence collection predominantly contained AY.30 sequences, while the non-BMR collection had a higher proportion of AY.85 sequences, suggestive of differing spatial distributions and transmission routes. In addition, the relationship between the estimated Ne values and reported weekly cases during the fourth wave differed significantly from those of other waves. Specifically, the estimated Ne values were found to be relatively low given the number of reported weekly cases in comparison to other waves (Fig. 4). This suggests that, since the vaccine coverage only began to become substantial after the middle of the fourth wave, there might have been a relatively small subset of viruses with high competitive advantage, especially those capable of evading COVID-19 vaccines and host immunity, that spread rapidly among a large number of hosts, resulting in less genetic diversity accumulation compared to other waves. Indeed, in addition to the omicrons, delta variants have also been reported to have an ability to escape from vaccine-induced immunity [59–62], albeit they might not be as good at this as the omicrons [59, 60]. Alternatively (although not mutually exclusive), there could have been a high sampling bias during the fourth wave, wherein the collected samples represented only a small portion of the true genetic diversity of the viruses. These findings underscore the need for a well-planned and systematic disease surveillance system for future outbreaks to avoid such potential biases. Plans for future disease response should also account for the possibility that pathogens of different variants might have differential spatial distributions as well as dynamic transmissibility and transmission routes.

In conclusion, despite the retrospective nature of this study, the information and results presented here provide valuable insights for enhancing and refining genomic surveillance practices for future emerging diseases in Thailand, and potentially in other developing countries.

## Supplementary Data

Supplementary material 1

Supplementary material 2
