# Supplementary material for: Spatiotemporal evolution of SARS-CoV-2 in the Bangkok metropolitan region, Thailand, 2020–2022: implications for future outbreak preparedness
Source: Microb Genom. 2023 Dec 20;9(12):001170. doi: 10.1099/mgen.0.001170 (PMC10763513; doi:10.1099/mgen.0.001170)
Supplement: Supplementary material 2 [file mgen-9-1170-s002.pdf]

Spatiotemporal evolution of SARS-CoV-2 in the Bangkok metropolitan region, Thailand, 2020—2022: Implications for future outbreak preparedness

Authors

Pakorn Aiewsakun[1,2]*, Bharkbhoom Jamsai[1,2], Worakorn Phumiphanjarphak[1,2], Waritta Sawaengdee [3], Prasit Palittapongarnpim[1,2], Surakameth Mahasirimongkol[3]*

Author affiliations

1. Department of Microbiology, Faculty of Science, Mahidol University, Bangkok, Thailand 10400
2. Pornchai Matangkasombut Center for Microbial Genomics, Department of Microbiology, Faculty of Science, Mahidol University, Bangkok, Thailand 10400
3. Genomic Medicine Centre, Division of Genomic Medicine and Innovation Support, Department of Medical Sciences, Ministry of Public Health, Nonthaburi 11000, Thailand

Corresponding authors' contact details

Pakorn Aiewsakun: Department of Microbiology, Faculty of Science, Mahidol University, 272, Rama VI Road, Ratchathewi, Bangkok, 10400, Thailand; +66 2 201 5543; pakorn.aie@mahidol.ac.th
Surakameth Mahasirimongkol: Department of Medical Sciences, Ministry of Public Health, 88/7 Tiwanon Road. Amphoe Muang, Nonthaburi, 11000, Thailand; +66 2 589 0022; surakameth.m@dmsc.mail.go.th

Supplementary Table

[See Supplementary Table 1.xlsx]

Supplemetary Table 1. Metadata of all sequences analysed in this study. The EPI_SET ID for the GISAID sequences analysed in this study is EPI_SET_231130hz (doi: 10.55876/gis8.231130hz)**.**

Supplementary Figures

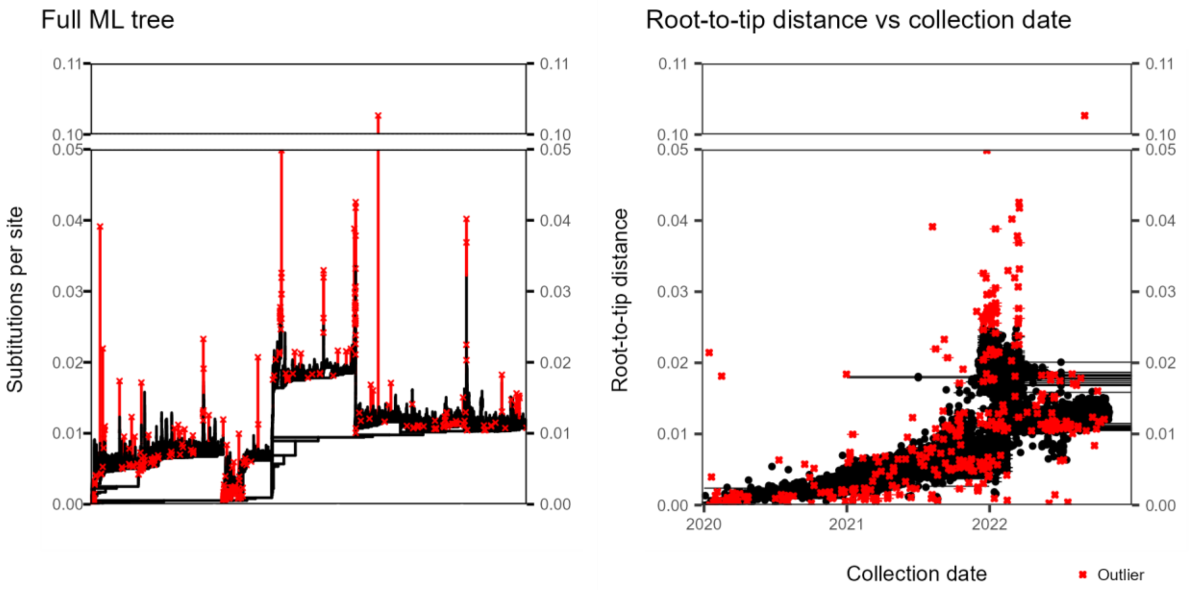

Supplementary figure 1 (Left) Maximum likelihood (ML) phylogeny of SARS-CoV-2 circulating in Thailand between 2020 and 2020 (n = 27,913) against a backdrop of global references (n = 7,329), and (right) the root-to-tip regression analysis. The ML phylogeny was estimated by using IQ-TREE2 v2.1.3 [1] under the GTR+I+Γ(4) nucleotide substitution model and the fast tree search mode (-fast). The Wuhan-Hu-1 sample, which was one of the earliest samples in the global COVID-19 pandemic, was used to root the tree. Red crosses indicate outliers, i.e. samples with atypical branch lengths compared to their collection times, as determined by lsd2 v2.4.1 [2] (n = 344, 0.98%). Horizontal lines in the root-to-tip regression analysis plot indicate collection date uncertainties.

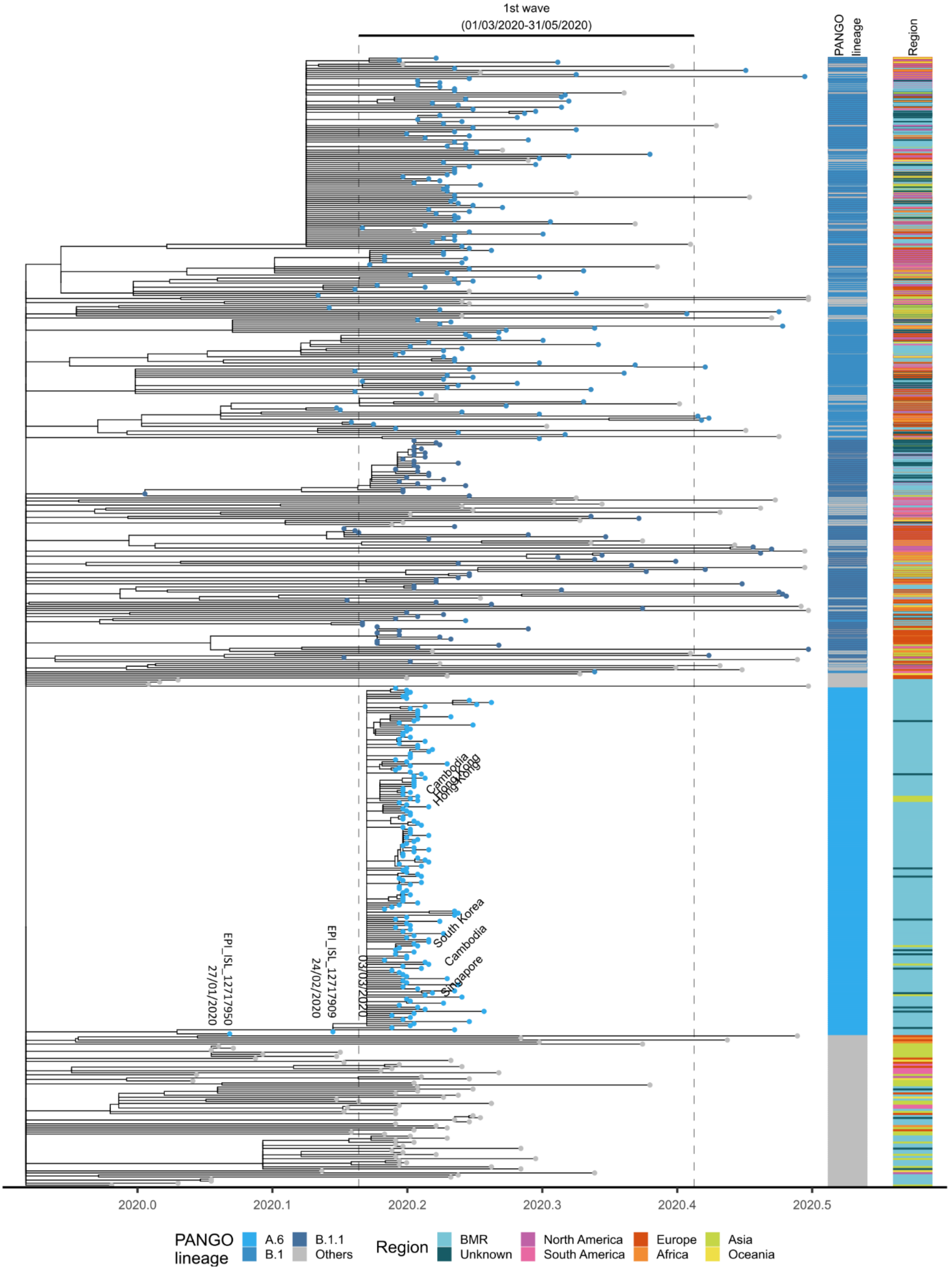

Supplementary Figure 2 Time-calibrated SARS-CoV-2 phylogeny focusing on the A.6, B.1, and B.1.1 variants, the dominant variants during the 1st COVID-19 wave in the BMR.

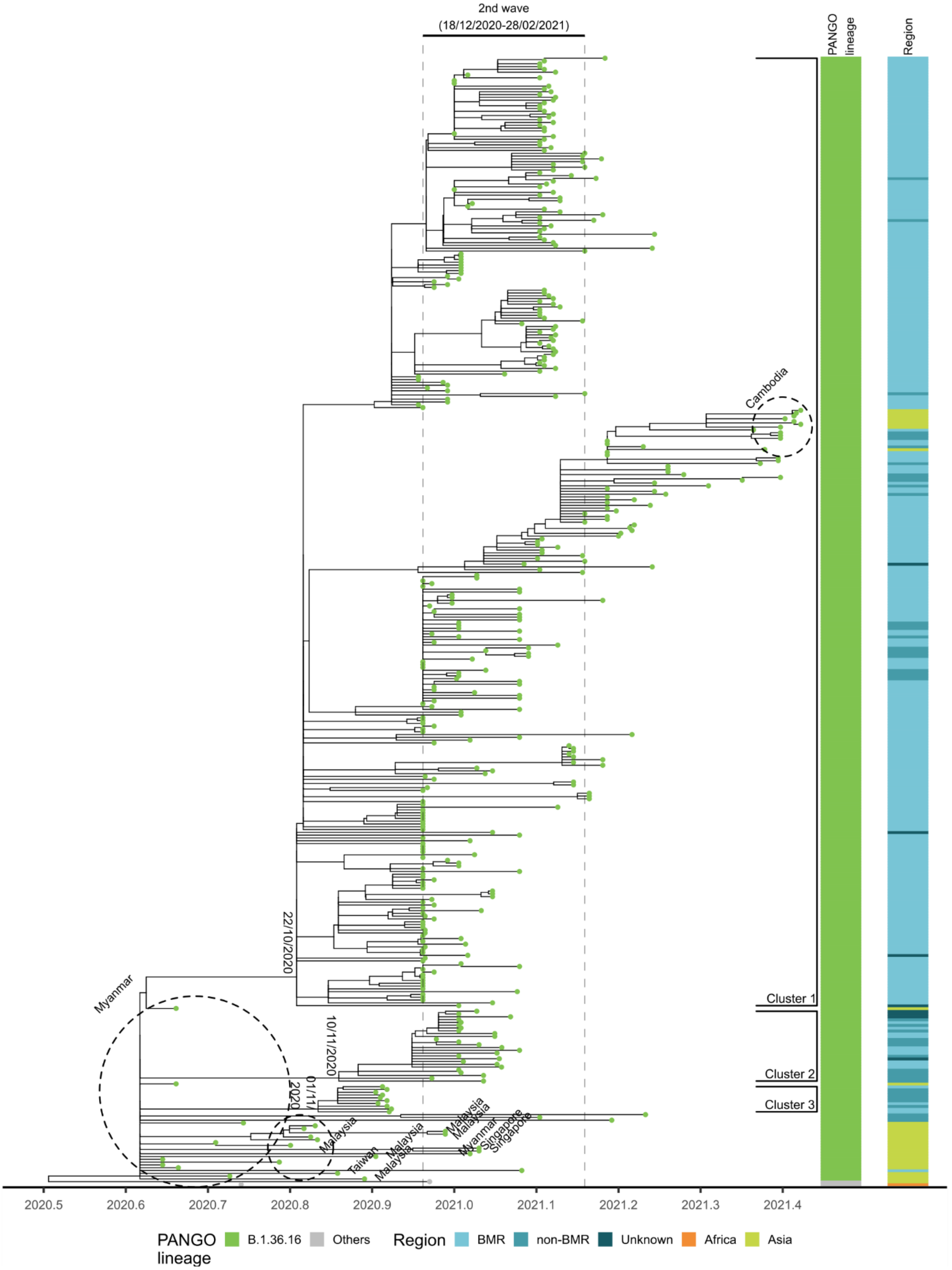

Supplementary Figure 3 Time-calibrated SARS-CoV-2 phylogeny focusing on the B.1.36.16 variant, the dominant variant during the 2nd COVID-19 wave in the BMR. Two B.1.36.16 samples with long terminal branches (EPI_ISL_13133216, and EPI_ISL_13133217) in Cluster 1 are not depicted on the figure.

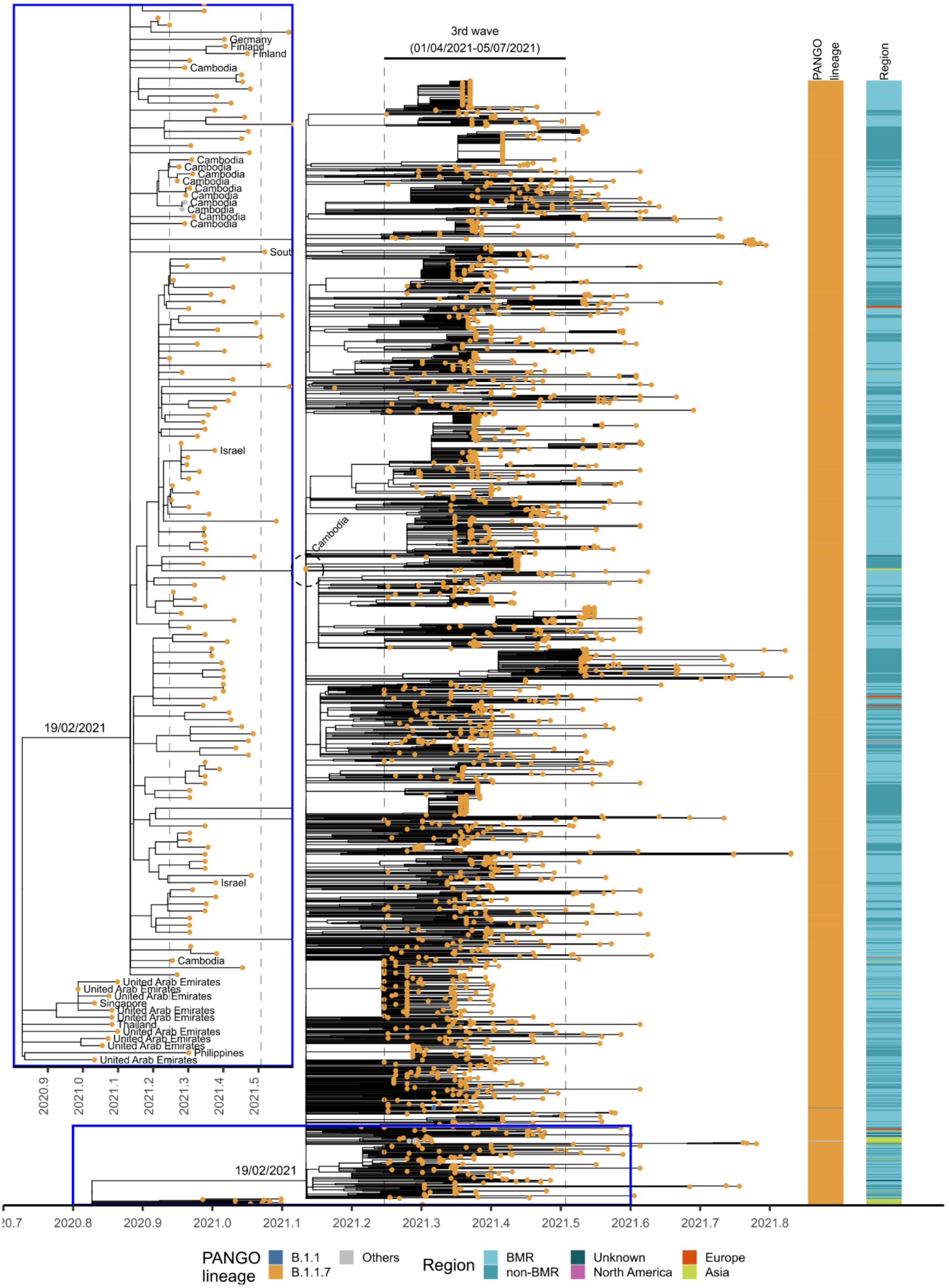

Supplementary Figure 4 Time-calibrated SARS-CoV-2 phylogeny focusing on the B.1.1.7 variant, the dominant variant during the 3rd COVID-19 wave in the BMR and Thailand.

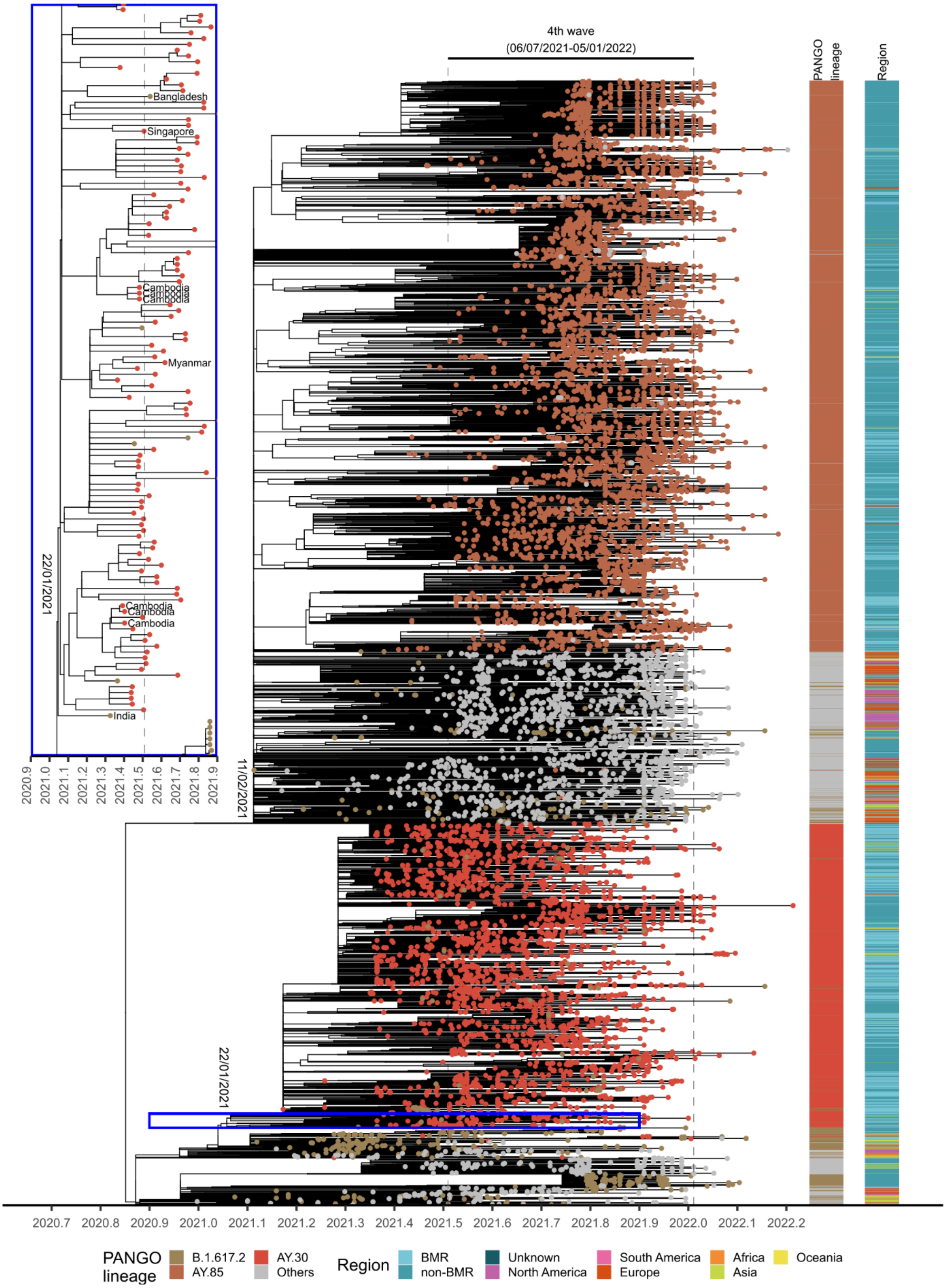

Supplementary Figure 5 Time-calibrated SARS-CoV-2 phylogeny focusing on the B.1.617.2, AY.30, and AY.85 variants, the dominant variants during the 4td COVID-19 wave in the BMR and Thailand. Seven samples of other sub-Delta variants with long terminal branches (OP778183, OP778178, OP778177, OP778181, OP583725, OP583739, and OP583740) are not shown on the figure.

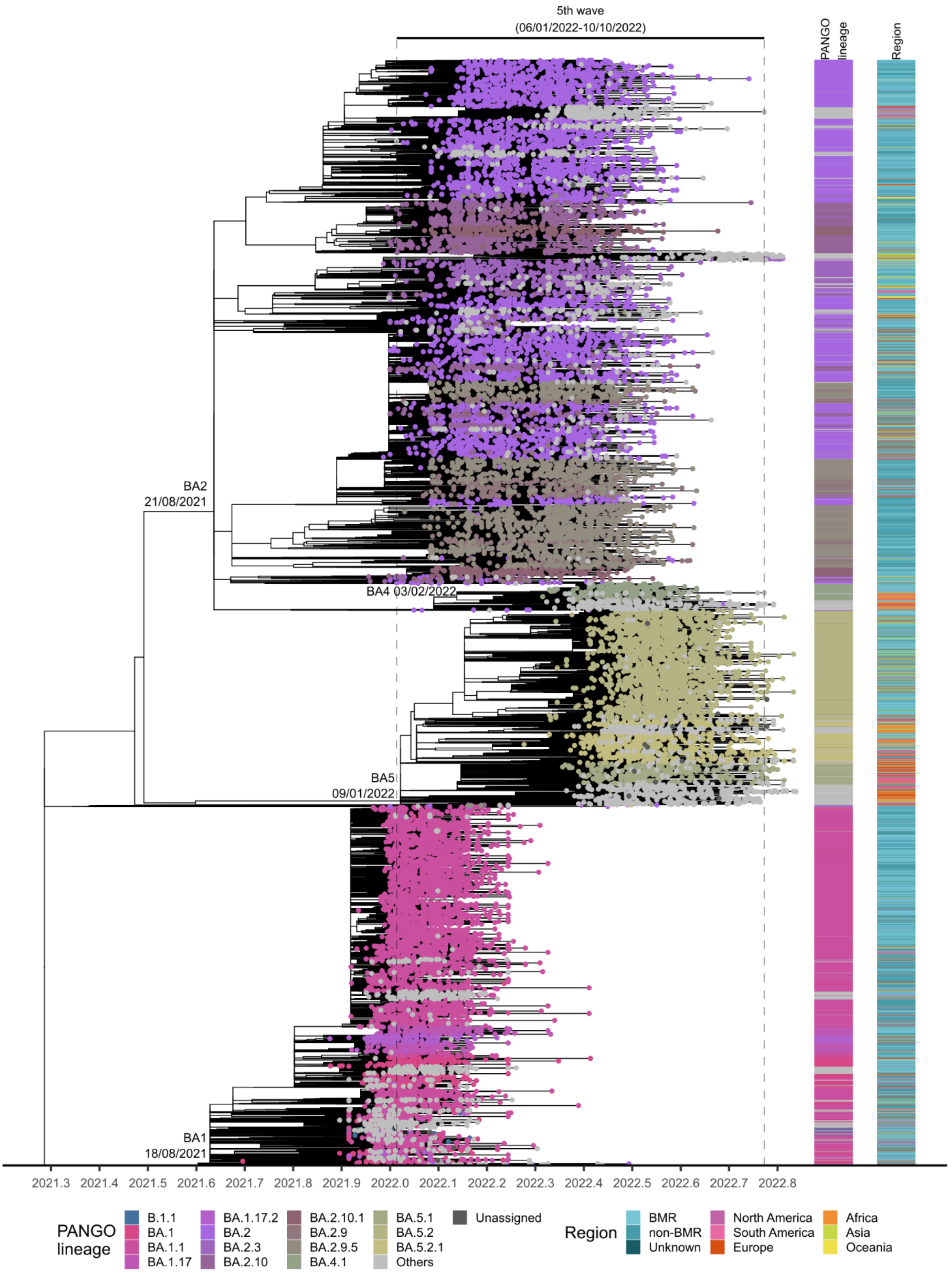

Supplementary Figure 6 Time-calibrated SARS-CoV-2 phylogeny focusing on the various sub Omicron variants, which dominated the 5th COVID-19 wave in the BMR and Thailand.

Supplementary Notes: Phylogenetic structures of individual virus variants dominating each COVID-19 wave in Thailand between 2020 and 2022

A.6, B.1 and B.1.1 – the dominant virus variants during the 1st wave

During the 1st wave of COVID-19, three dominant SARS-CoV-2 variants were detected in the BMR, namely A.6, B.1 and B.1.1 (**Figure 2, main text**). A.6 was found to be monophyletic, comprising primarily viruses from the BMR, along only a few sequences from other Asian countries, including Cambodia (2 sequences), Hongkong (2), South Korea (1), and Singapore (1) (**Supplementary Figure 2**). This pattern indicates that all of the A.6 BMR sequences in the dataset were sampled from a single outbreak sharing a single origin, and the outbreaks was mainly restricted to the BMR. The estimated time to most recent common ancestor (tMRCA) of the main A.6 cluster was ~2020.17 (03/03/2020), about a week before the earliest sequence within the cluster (09/03/2020). However, basal to the main cluster were two A.6 sequences from Nonthaburi, which is also part of the BMR, reported in late January (27/01/2020), and late February (24/02/2020). This suggests that the virus might have already been present in the BMR by then but without being noticed.

B.1 and B.1.1 sequences, in contrast, were found to be non-monophyletic, scattering across the base of the tree. In particular, they were found to be basal to, and / or cluster with, the B.1.1.7 (Alpha) variant, and B.1.1.529 and BA (Omicron) variants (**Figure 3, main text**). This is not surprising however, given that B.1.1.7, B.1.1.529 and BA evolved from B.1 and B.1.1 variants by definition. In addition, BMR B.1 and B.1.1 sequences were found to cluster together with sequences reported from outside Thailand but without forming large clusters (**Supplementary Figure 2**). These sequences likely represented sporadic imported cases that Thai authorities effectively identified, quarantined, and thereby prevented them from causing significant outbreaks within the region.

B.1.36.16 – the dominant virus variant during the 2nd wave

B.1.36.16 was found to be most prevalent during the 2nd wave (**Figure 2, main text**). Three clusters of Thai B.1.36.16 sequences were found with comparable tMRCAs, falling between late October and early November (Cluster 1: ~2020.808, 22/10/2020; Cluster 2: ~2020.859, 10/11/2020; Cluster 3: ~2020.834, 01/11/2020); however, only one cluster (Cluster 1) appeared to dominate the outbreak (**Supplementary Figure 3**). The earliest Thai sequence within these 3 clusters was collected on 28/11/2020, suggesting a possibility that the virus had likely been circulating in the country for about a month (or more) before causing large outbreaks.

While the majority of the sequences in the clusters were from the BMR, they also contained a few sequences from other areas of Thailand, as well as some from Cambodia forming a tight clade (**Supplementary Figure 3**). This phylogenetic pattern suggests that there were a few sporadic transmissions of the virus from the BMR to other areas of Thailand during the 2nd wave, as well as at least one instance of virus exportation from Thailand to Cambodia. Immediately basal to the Thai B.1.36.16 cluster are B.1.36.16 sequences from Myanmar. This observation supports potential epidemiological connections between Thailand and Myanmar in the early stages of the outbreak, previously speculated based on epidemiological profiles of the virus and patients during that time [3].

B.1.1.7 – the dominant virus variant during the 3rd wave

The B.1.1.7 (Alpha) variant was the dominant variant during the first half of the 3rd COVID-19 wave in the BMR (**Figure 2, main text**), and their sequences formed a single large cluster on the tree (**Supplementary Figure 4**). Unlike the A.6 and B.1.36.16 variants, while the cluster still contained only a few sequences reported from outside Thailand, it contained a relatively large number of non-

BMR sequences (n = 805), and they phylogenetically intermixed with the BMR sequences (n = 1,252). These results are consistent with that these B.1.1.7 sequences were collected from a single large outbreak in Thailand, and the wide-spread of the virus in the country was likely due to extensive local transmissions of the virus, rather than multiple introductions of the virus into the country.

Their MRCA was dated to around 2021.134 (19/02/2021), about two weeks before the earliest Thai sequence in the clade (06/03/2021). Notably, precisely at the base of the clade were 3 Cambodian sequences collected on 19/02/2021 (**Supplementary Figure 4, dotted circle**), and there were 12 more Cambodian sequences collected between 04-26/04/2021 clustering with Thai sequences (**Supplementary Figure 4, inset figure**). Moreover, the sister clade of the Thai B.1.1.7 sequence cluster was a clade of sequences reported from the United Arab Emirates collected between 27/12/2020 – 06/02/2021, which also contained 1 Singaporean sequence (13/01/2021), 1 Thai sequence (01/2021), and 1 Philippine sequence (21/04/2021) (**Supplementary Figure 4, inset figure**). This finding suggests potential epidemiological connections between Thailand and these countries in the early stages of the outbreak.

B.1.617.2, AY.30, and AY.85 – the dominant virus variants during the 4th wave

The classic Delta B.1.617.2 variant, and its two subvariants, including AY.30, and AY.85 variants, began appearing in the BMR since the middle of the 3rd wave (**Figure 2, main text**). The three virus variants gradually and steadily replaced the B.1.1.7 variant, and became the predominant variants in the region, eventually causing the 4th wave of COVID-19 in Thailand. Phylogenetic analysis revealed that AY.30, and AY.85 formed their own distinct clusters, and B.1.617.2 sequences scattered around the base of the two (**Supplementary Figure 5**). This pattern was expected, as AY.30 and AY.85 are by definition within the diversity of the B.1.617.2 variant.

Analyses showed that, similar to the B.1.1.7 cluster, both AY.30 and AY.85 clusters consisted mainly of sequences from Thailand, but both had relatively large numbers of BMR and non-BMR sequences that were phylogenetically intermixing with each other (AY.30: n_{BMR} = 1,319, $n_{non\text{-}BMR}$ = 1,290; AY.85: n_{BMR} = 1,021, $n_{non\text{-}BMR}$ = 4,024, **Supplementary Figure 5**). Logistic regression analysis revealed a significant difference in the proportion of AY.30 to AY.85 sequences between the BMR and non-BMR sequence collections (Type III ANOVA test: df = 1, χ^2 = 725.47, $p < 2.2\times10^{-16}$), with the former showing a greater proportion of AY.30 (56.4%, 95% confidence interval = 54.3–58.4%) compared to the latter (24.3%, 95% confidence interval = 23.1–25.4%).

The two clusters were not sister groups on the tree, and basal to them were different sets of non-Thai sequences, indicating independent introductions of the viruses to the country. The MRCAs of the AY.30 and AY.85 variants were dated to ~2021.058 (22/01/2021), and ~2021.112 (11/02/2021), respectively. This suggests that the two variants likely emerged months before causing larger outbreaks in Thailand. In addition, while the base of the cluster of AY.85 was not well resolved, we found that positioning immediately at the base of the AY.30 cluster were a few sequences from Cambodia (6), Myanmar (1), Singapore (1), and Bangladesh (1) (**Supplementary Figure 5, inset**), suggestive of epidemiological linkages between Thailand and these countries in the early phases of the outbreak.

BA variants – the dominant virus variants during the 5th wave

Numerous sub Omicron variants were detected in the sequence data collected during the 5th wave, but which could be tentatively grouped into four major variants, including BA.1, BA.2, BA.4, and BA.5 (**Figure 2, main text**), each forming their own major sequence phylogenetic cluster (**Supplementary Figure 6**).

Analysis revealed that, unlike other variants described above, both Thai and non-Thai Omicron sequences intermixed together on the tree in all four major Omicron clusters, as well as sequences from the BMR and other regions of the country (**Supplementary Figure 6**). This pattern supports that the 5th wave of COVID-19 in Thailand was a result of multiple independent virus introductions, coupled with extensive local transmissions. This result, again, underscores Thailand, particularly the BMR, as a hotspot for emerging infectious diseases.

The tMRCAs for BA.1 and BA.2 variants were both estimated to be in August 2021 (BA.1: ~2021.628, 18/08/2021, and BA.2: ~2021.636, 21/08/2021). For BA.4 (mostly BA.4.1) and BA.5, their tMRCAs were estimated to be in early 2022 (BA.4: ~2022.090, 03/02/2022; and BA.5: ~2022.022, 09/01/2022). However, given each cluster contained numerous non-Thai sequences, these date estimates likely represent the tMRCAs of the viruses' themselves rather than viruses in Thailand.

References

1. **Minh BQ, Schmidt HA, Chernomor O, Schrempf D, Woodhams MD, *et al.*** IQ-TREE 2: New models and efficient methods for phylogenetic inference in the genomic era. *Mol Biol Evol* 2020;37:1530–1534.

2. **To TH, Jung M, Lycett S, Gascuel O**. Fast dating using least-squares criteria and algorithms. *Syst Biol* 2016;65:82–97.

3. **Rajatanavin N, Tuangratananon T, Suphanchaimat R, Tangcharoensathien V**. Responding to the COVID-19 second wave in Thailand by diversifying and adapting lessons from the first wave. *BMJ Glob Heal* 2021;6:e006178.
